# Editorial: Emerging challenges in companion animal toxicology

**DOI:** 10.3389/fvets.2025.1610134

**Published:** 2025-05-01

**Authors:** Nicola Bates, Lynn Rolland Hovda, Kristin Opdal Seljetun

**Affiliations:** ^1^Veterinary Poisons Information Service, London, United Kingdom; ^2^Safetycall International and Pet Poison Helpline, Minneapolis, MN, United States; ^3^Norwegian Scientific Committee for Food and Environment, Norwegian Institute of Public Health, Oslo, Norway

**Keywords:** pets, poisoning, small animals, toxicity, dogs

Companion animals share our homes and can be exposed to a wide variety of potentially harmful substances such as plants, mushrooms, household products, chemicals, pesticides, and human and veterinary medicines. Despite the daily exposure of pets to potentially toxic products, the incidence of case studies of animal poisoning is relatively low compared to other causes of illness. This discrepancy presents a challenge for veterinary practitioners, as they may be presented with many different clinical toxicologic scenarios.

This Research Topic “*Emerging challenges in companion animal toxicology*” presents eight manuscripts that improve our knowledge and understanding of poisoning agents and treatment protocols.

Initial management of poisoning in veterinary cases usually involves gastric decontamination, typically induction of emesis and administration of activated charcoal as an adsorbent. Apomorphine, a dopamine agonist, has long been the main emetic used in dogs, but the novel dopamine-2 specific agonist ropinirole has also been licensed for this indication. Lee J. A. et al. evaluated the effect of ophthalmic ropinirole vs. intravenous apomorphine in dogs. Their study found ropinirole to be non-inferior to apomorphine. Ropinirole had a longer time to onset of action and more prolonged vomiting but had a similar incidence of adverse effects and efficacy compared to apomorphine. Activated charcoal is another commonly used treatment for small animal poisoning, due to its ability to bind to a variety of toxicants (1, 2). Activated charcoal binds to compounds in the gastrointestinal tract, resulting in decreased adsorption, thus reducing or preventing systemic toxicity (3). Hypernatremia is a recognized adverse effect of charcoal administration and Young et al. carried out a retrospective study from 2018 to 2023 on the incidence of hypernatremia after such treatment. Their research found that administration of a single dose of activated charcoal, with or without co-administration of a cathartic, was not associated with the development of hypernatremia in dogs with acute toxicant ingestion.

Intravenous lipid emulsion (ILE) is used in the management of poisoning with a number of different substances, usually lipophilic compounds but also cardiotoxic substances. The mechanism of action of ILE is not fully understood but is commonly described as a “lipid sink” or “shuttle” (4). The study by Jones et al. examined the effect of ILE on the blood concentrations of baclofen, ibuprofen, and bromethalin in dogs. ILE therapy was only effective in reducing bromethalin concentrations, supporting the lipid sink theory, and it was concluded that ILE therapy may have other means of significantly reducing lipophilic drug concentrations in cases of toxicosis.

Other studies and case reports presented within this Research Topic demonstrate the variety of potentially harmful substances in our environment. Natural toxins pose a potential risk, such as stinging insects. The case report by Lee J.-M. et al. described multiple organ failure in a small dog following multiple stings by a paper wasp. Cases of multiple stings by bees and wasps have been reported in dogs but this is the first report involving paper wasps.

In another case report, McDermott et al. described a case of thallium poisoning in a dog and this case serves as a reminder that substances that are no longer in use still pose a risk to pets if not stored or disposed of safely. In this case the national poison center played an essential role in providing advice on management and access to the antidote, Prussian Blue. Poison centers are a vital resource in the management of veterinary and human poisonings. The World Health Organization states that ‘poison centers are sources of specialized expertise to address the fact that health professionals could not be expected to know about the toxicity of every chemical substance and product and also to provide a focus for toxicological research' (5). This is equally true for veterinary and human poison centers.

Petronzio et al. alerted us to the risk of side effects from therapeutic doses of medications. Their case report described a case of suspected bone marrow suppression after administration of febantel, a compound that is metabolized to fenbendazole and oxyfenbendazole. The previously healthy dog was treated with febantel in combination with praziquantel and pyrantel pamoate prescribed for empiric treatment of giardia, although the dog was neither symptomatic nor fecal antigen positive. Bone marrow suppression has been reported with fenbendazole and albendazole, another benzimidazole anthelmintic, but not with febantel. Other causes of bone marrow toxicity were excluded and the authors suggested that metabolism to fenbendazole, an active metabolite, resulted in an idiosyncratic drug reaction. This case serves as a reminder that adverse effects can occur with commonly used medications even when dosed appropriately.

The article by Klainbart et al. demonstrated the increasing trend of exposure to illicit drugs in companion animals. The study described confirmed cases of drug abuse exposure in pets from a California laboratory and found that the most common drugs were amphetamine-type stimulants. However, in nearly half of the cases, more than one drug was found and a number of cases involved animal cruelty. The research shed light on the malicious poisoning of animals and the need for further study in this area.

Ingestion of human medications in dogs is a frequent call to animal poison centers (6, 7). The review by Lohmann-Menezes et al. highlighted the importance of species differences in both therapeutic usage and risk of toxicosis using the example of the non-steroidal anti-inflammatory drug (NSAID) diclofenac in dogs. The study emphasized the importance of seeking veterinary advice before administering drugs to companion animals.

In summary, the articles included in this Research Topic illustrate some of the circumstances under which poisoning of companion animals can occur. All of these studies highlight the wide variety and complexity of cases that emergency veterinary clinicians may encounter, and together they contribute to our knowledge of the risks and management of poisoning in companion animals.
